# Insecticidal Activity of Four Essential Oils Extracted from Chilean Patagonian Plants as Potential Organic Pesticides

**DOI:** 10.3390/plants11152012

**Published:** 2022-08-02

**Authors:** Cristiano Giordani, Eleonora Spinozzi, Cecilia Baldassarri, Marta Ferrati, Loredana Cappellacci, Daniela Santibañez Nieto, Roman Pavela, Renato Ricciardi, Giovanni Benelli, Riccardo Petrelli, Filippo Maggi

**Affiliations:** 1 Instituto de Física, Facultad de Ciencias Exactas y Naturales, Universidad de Antioquia, Calle 70 No 52-21, Medellín 050010, Colombia; cristiano.giordani@udea.edu.co; 2 Grupo Productos Naturales Marinos, Facultad de Ciencias Farmacéuticas y Alimentarias, Universidad de Antioquia, Calle 70 No 52-21, Medellín 050010, Colombia; 3 School of Pharmacy, University of Camerino, Chemistry Interdisciplinary Project (ChIP), via Madonna delle Carceri, 62032 Camerino, Italy; eleonora.spinozzi@unicam.it (E.S.); cecilia.baldassarri@unicam.it (C.B.); marta.ferrati@unicam.it (M.F.); loredana.cappellacci@unicam.it (L.C.); riccardo.petrelli@unicam.it (R.P.); filippo.maggi@unicam.it (F.M.); 4 Productos Walwalün, Valle Mirta, La Junta, Property Walwalün, Región de Aysén 6019105, Chile; d2niets@hotmail.com; 5 Crop Research Institute, Drnovska 507, 16106 Prague, Czech Republic; pavela@vurv.cz; 6 Department of Plant Protection, Czech University of Life Sciences Prague, Kamycka 129, 16500 Praha, Czech Republic; 7 Department of Agriculture, Food and Environment, University of Pisa, via Del Borghetto 80, 856124 Pisa, Italy; renato.ricciardi@unipi.it

**Keywords:** bioinsecticide, green insecticide, *Culex quinquefasciatus*, *Musca domestica*, *Spodoptera littoralis*, contact toxicity, mosquito, moth, housefly

## Abstract

Patagonia is a geographical area characterized by a wide plant biodiversity. Several native plant species are traditionally used in medicine by the local population and demonstrated to be sources of biologically active compounds. Due to the massive need for green and sustainable pesticides, this study was conducted to evaluate the insecticidal activity of essential oils (EOs) from understudied plants growing in this propitious area. Ciprés (*Pilgerodendron uviferum*), tepa (*Laureliopsis philippiana*), canelo (*Drimys winteri*), and paramela (*Adesmia boronioides*) EOs were extracted through steam distillation, and their compositions were analyzed through GC–MS analysis. EO contact toxicity against *Musca domestica* L., *Spodoptera littoralis* (Boisd.), and *Culex quinquefasciatus* Say was then evaluated. As a general trend, EOs performed better on housefly males over females. Ciprés EO showed the highest insecticidal efficacy. The LD_50(90)_ values were 68.6 (183.7) and 11.3 (75.1) µg adult^−1^ on housefly females and males, respectively. All EOs were effective against *S. littoralis* larvae; LD_50_ values were 33.2–66.7 µg larva^−1^, and tepa EO was the most effective in terms of LD_90_ (i.e., <100 µg larva^−1^). Canelo, tepa, and paramela EOs were highly effective on *C. quinquefasciatus* larvae, with LC_50_ values < 100 µL L^−1^. Again, tepa EO achieved LD_90_ < 100 µL L^−1^. This EO was characterized by safrole (43.1%), linalool (27.9%), and methyl eugenol (6.9%) as major constituents. Overall, Patagonian native plant EOs can represent a valid resource for local stakeholders, to develop effective insecticides for pest and vector management, pending a proper focus on their formulation and nontarget effects.

## 1. Introduction

Patagonia is a geographical region in the world’s southern hemisphere shared by Chile and Argentina. A part of this region belongs to one of the 35 world biodiversity hotspots, places of plant endemic biodiversity. In the hotspot called “Central Chile”, there are about four thousand native plant species, half of which are endemic and turn out to be a rich source of biologically active compounds. Between this wide biodiversity, paramela (*Adesmia boronioides* Hook.f.), canelo (*Drimys winteri* J.R.Forst. & G.Forst.), tepa (*Laureliopsis philippiana* (Looser) Schodde), and ciprés de las Guaitecas (*Pilgerodendron uviferum* (D. Don) Florin) have a well-recognized role in Patagonian traditional medicine and culture, finding large interest for their numerous biological activities.

*A. boronioides*, also known as paramela, is an aromatic and medicinal species belonging to the Fabaceae family [1,2]. It is a resinous shrub, 0.40 to 2 m high, which has been used to treat rheumatic pain and hair loss [3], as incense for the respiratory tract, as a digestive [4], as an aphrodisiac, and to alleviate menstrual discomfort [5]. This species has received an increasing interest, especially for its essential oil (EO), which has been reported for its antimicrobial, antifungal, trypanocidal, and anti-inflammatory activities [6,7].

A second noteworthy species is *L. philippiana*, known as tepa or huanhuán [8], which belongs to the Atherospermataceae family [9]. It is traditionally used as a decongestant and antibiotic agent, bronchodilator, anti-allergenic, anti-inflammatory, energizing, immunostimulant, analgesic, and to calm inflammation of varicose veins [10,11]. Recently, tepa EO fumigant insecticidal activity and the repellent and antifeedant effects have been reported as promising on adults of *Sitophilus zeamais* Motsch, *Sitophilus oryzae* L., and *Sitophilus granarius* L. (Coleoptera: Dryophthoridae) [12,13].

Another interesting species found in the Patagonian region is *D. winteri,* commonly known as canelo, which belongs to the Winteraceae family. This shrub has been used for the treatment of rheumatism, skin infections, inflammation, gastrointestinal problems, colds, and hypertension [14]. Recently, the bioactivity of canelo EO against *Acyrthosiphon pisum* (Harris) (Hemiptera: Aphididae) aphids has been investigated [15]; it has shown insecticidal effects against *Acanthoscelides obtectus* Say (Coleoptera: Bruchidae) and *Aegorhinus superciliosus* Guérin (Coleoptera: Curculionidae) [16].

Lastly, *P. uviferum*, also known as the ciprés de las Guaitecas, belongs to the Cupressaceae family [9] and is an endemic tree reaching diameters of up to 1.1 m and heights of more than 20 m [17]. The ciprés of the Guaitecas is used externally as a medicinal ointment for treating lumbar pain, stress, and varicose veins. *P. uviferum* EO is considered a good model in the search for raspberry weevil repellents [18], and it showed an effectiveness in reducing the adult growth of *Hylastinus obscurus* Marsham (Coleoptera: Curculionidae) [19].

The aim of this work was to investigate the insecticidal potential of the EOs from these Patagonian plants against adults of *Musca domestica* L. (Diptera: Muscidae), and third-instar larvae of *Spodoptera littoralis* Boisd. (Lepidoptera: Noctuidae) and *Culex quinquefasciatus* Say (Diptera: Culicidae). Given the need to manage invasive and dangerous arthropod vectors and pests [20], coupled with the importance to face the increasing insecticide resistance with alternative green and sustainable pesticides [21,22,23], these endemic and under-researched plants may represent a source of potential insecticidal products and, consequently, a chance for economic development for the region’s economy.

## 2. Results

### 2.1. Chemical Characterization of Essential Oils

Table 1 shows the results obtained from the GC–MS analysis of the four EOs. Ciprés EO was composed mainly of sesquiterpene hydrocarbons (81.2%) and oxygenated sesquiterpenes (17.2%), accounting for 98.3% of the total composition. The most abundant compounds were *δ*-cadinene (44.9%), *trans*-cadina-1(6),4-diene (8.3%), 1-*epi*-cubenol (7.3%), and *α*-copaene (6.1%). Other constituents were cubenol (5.4%), (*E*)-caryophyllene (4.9%), *α*-humulene (3.9%), *trans*-calamenene (3.4%), *α*-muurolene (2.9%), and *α*-calacorene (2.4%). Low concentrations of *trans*-cadina-1,4-diene (1.6%), gleenol (1.5%), *α*-muurolol (1.4%), *γ*-muurolene (0.8%), epizonarene (0.6%), and *α*-eudesmol (0.6%) were also detected.

Tepa EO presented a different composition profile, being mainly constituted by phenylpropanoids (51.1%), with a dominance of safrole (43.1%) and methyl-eugenol (6.9%). Oxygenated monoterpenes and monoterpene hydrocarbons were also important fractions of this EO, with percentages of 30.3 and 18.0%, respectively. Among them, linalool (27.9%) and 1,8-cineole (8.5%), and *α*-phellandrene (3.0%), *β*-pinene (1.3%), and *p*-cymene (1.1%) were the most representative compounds, respectively.

Canelo EO obtained from *D. winteri* appeared to be mainly constituted by monoterpene hydrocarbons (48.7%) and oxygenated sesquiterpenes (39.0%). Lower concentrations of oxygenated monoterpenes and sesquiterpenes hydrocarbons were also detected, with percentages of 4.4 and 5.9%, respectively. Among monoterpene hydrocarbons, *α*-pinene and *β*-pinene were the most abundant ones (18.8% and 21.5%, respectively), whereas hedycaryol (18.2%), *α*-eudesmol (6.3%), *β*-eudesmol (5.2%), and *γ*-eudesmol (6.6%) have been found as the most representative of the sesquiterpene class.

Paramela EO from *A. boronioides* has been found to be mainly composed of oxygenated sesquiterpenes (57.2%) and monoterpene hydrocarbons (13.6%), accounting for 79.9% of the total composition. Oxygenated monoterpenes and sesquiterpene hydrocarbons were also detected in lower concentrations (3.5 and 5.3%, respectively). In detail, the principal compounds identified were the oxygenated sesquiterpenes esquel-6-en-9-one (30.7%) and esquel-7-en-9-one (10.2%).

**Figure 1:**
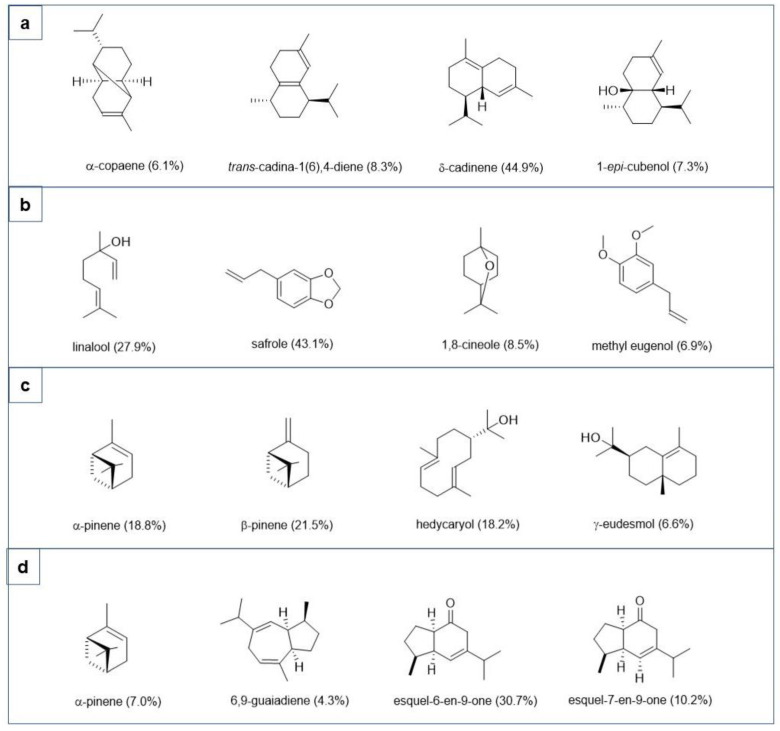
Main bioactive compounds of ciprés (**a**), tepa (**b**), canelo (**c**), and paramela (**d**) essential oils.

Figure 1 shows the chemical structure of the main bioactive compounds of ciprés, tepa, canelo and paramela EOs.

### 2.2. Insecticidal Activity

#### 2.2.1. Insecticidal Activity against Houseflies

The tested EOs provided promising efficacy against *M. domestica* adults (Table 2). Generally, it can be noted that males showed a significantly higher sensitivity; significantly lower lethal doses were estimated for them (except for tepa EO), if compared with females. On the other hand, despite the significant difference in efficacy between the housefly sexes, no difference was observed in terms of efficacy between individual EOs, as the confidence intervals overlapped at least in one LD_50_ parameter in each case.

Nevertheless, considering the lowest LD_50(90)_ values, the following two EOs provided the best results: ciprés EO, with LD_50(90)_ estimated as 68.6 (183.7) and 11.3 (75.1) µg adult^−1^ for females and males, respectively, and paramela EO, with LD_50(90)_ estimated as 65.2 (195.1) and 11.1 (113.1) µg adult^−1^ for females and males, respectively.

#### 2.2.2. Insecticidal Activity against Moths

The efficacy of EOs in terms of acute toxicity for *S. littoralis* larvae is presented in Table 3. All EOs provided promising efficacy; their LD_50_ values ranged from 33.8 to 66.7 µg larva^−1^, while LD_90_ values ranged from 72.3 to 124.5 µg larva^−1^. Nevertheless, tepa EO provided the highest efficacy where the confidence interval for LD_90_ was estimated as less than 100 µg larva^−1^.

#### 2.2.3. Insecticidal Activity against Mosquitoes

Significant differences in the EO efficacy were observed on *C. quinquefasciatus* larvae (Table 4). In terms of mosquito insecticidal efficacy, highly promising EOs were canelo, tepa, and paramela, with LC_50_ values estimated as less than 100 µL L^−1^.

Tepa EO was the most efficient one with the confidence interval for LD_90_ estimated as less than 100 µL L^−1^.

## 3. Discussion

In general, the composition of the EOs from ciprés, tepa, canelo, and paramela are strongly influenced by the geographic area of distribution within Patagonia. Indeed, concerning ciprés EO, in a study conducted by Malizia et al. [24], the analyzed EO was obtained by plants collected in Argentinian Patagonia spontaneous forests and was mainly constituted by monoterpenes (54.1%), which instead are present only in traces in the EO analyzed in our study, although with a large presence of sesquiterpenes (40.4%). The sesquiterpene nature of the EO presented in this work is consistent with the ones reported by Oyarzun and Garbarino [25] and Espinoza et al. [18]; in both studies, the analyzed EOs were obtained from Chilean varieties of *P. uviferum*. However, differences in the main constituents of the EO have been detected: *δ*-cadinene and *α*-copaene were present in a lower amount (10.8 and 0.7%, respectively), while cubenol, which was present in a low concentration, was the most abundant compound (22.6%) [18,19], suggesting that the difference in composition can be due to other factors such as environmental stress and season of collection.

On the other hand, the usual composition of tepa EO is in accordance with previous studies from Norambuena et al. [13] and Madrid et al. [26], in which phenylpropanoid compounds are the most abundant class, with 78.4 and 67.6%, respectively, with safrole and methyl eugenol as the major compounds, though with different concentrations (17.0% and 24.4% for safrole and 61.4% and 7.12% for methyl eugenol, respectively). At the same time, in two independent studies, no sesquiterpenes were detected from the GC–MS analysis of the EO, and monoterpenes were the dominant class of compounds, with 1,8-cineole (13.89–37.4%) and linalool (32.3%) resulting as the principal compounds [10,11]. These differences in the composition may be related to the field collection season and the trees’ geographical area [13].

*α*-Pinene and *β*-pinene are the most abundant constituents of canelo EO in accordance with data reported in the literature [16,27,28,29], although in different proportions. In the study from Barrero et al. [27], both *α*-pinene and *β*-pinene were present in lower percentages than those found in the presented work (14.9 and 5.9%, respectively). From the analysis of Monsalvez et al. [28], *α*-pinene was the most abundant compound with a percentage of 71.2%, while *β*-pinene had a lower concentration (14.2%). The composition of α-pinene and β-pinene is likely dependent on the collection area. In fact, in a study conducted by Muñoz et al. [29], both insular and continental *D. winteri* EOs were analyzed, resulting in a different monoterpene profile; the EO obtained from plants collected in Chiloé island (southern Chile) was constituted by high levels of monoterpene hydrocarbons (92%), particularly *α*-pinene (23.1%) and *β*-pinene (43.6%), while the EO from plants collected in the metropolitan region of Santiago (central Chile) presented much lower *α*-pinene and *β*-pinene percentages (2.9 and 1.3%, respectively) and higher percentages of sesquiterpenes (32%) and phenylpropanoids (27%). This tendency was confirmed by other studies in which the material was collected in different regions. For example, Verdeguer et al. [30] analyzed a *D. winteri* EO obtained from plants collected in the V region of central Chile and found out that the percentage of monoterpenes was very low (*β*-pinene had a percentage of 2.7%, while *α*-pinene was completely absent). On the other side, Monsalvez et al. [28] obtained their EO rich in monoterpenes by collecting the plant material in the Nǔble Province of Chile, in the southern regions.

Paramela EO extracted from *A. boronioides* was mainly characterized by the presence of esquel-6-en-9-one and esquel-7-en-9-one. In our analysis, these compounds were identified only through the matching of the mass fragmentation with MS-spectral libraries. Their actual presence was then confirmed by data reported in the literature [31], indicating the presence of esquel-6-en-9-one and esquel-7-en-9-one in percentages of 19.1 and 12.5%, respectively. In addition, *α*-pinene was detected in the study by Gonzalez et al. [32] even though this monoterpene was less abundant than in the present study (3%). The composition of *A. boronioides* is susceptible to various conditions, as reported by Gonzalez et al. [7]. The sesquiterpene class remains the principal one characterizing this EO.

EOs are complex mixtures, even of several dozens of compounds; several factors can have a significant impact on their insecticidal efficacy, including their chemical composition [33], mutual synergistic relationships among the EO constituents [34,35], the mechanisms of action of active substances [33], and the mode of application and post-application conditions [36,37]. Regarding the tested EOs, it can be noted that they were very complex mixtures where the content of none of the major compounds was higher than 50%. Thus, it is difficult to determine which of the compounds was responsible for the highest biological activity as not only the above-mentioned synergistic, but also antagonistic relationships between the present substances may have played a role and may have reduced the final insecticidal efficacy [34,35].

This is the first report on the insecticidal efficacy of EOs obtained from these plant species against *M. domestica*, *S. littoralis,* and *C. quinquefasciatus*. However, some of these EOs have already been tested for insecticidal efficacy against other target organisms. For example, the EO from *P. uviferum* with *δ*-cadinol (24.16%), cubenol (22.64%), 15-copaenol (15.46%), and *δ*-cadinene (10.81%) as the major compounds has been tested against *Haematobia irritans* (L.) (Diptera: Muscidae) [38]. The authors reported very good insecticidal efficacy in their fumigation tests with LC_50_ values for *P. uviferum* EO of 9.41 and 1.02 µL L^−1^ air at 1 and 4 h, respectively. The authors also found a promising repellent efficacy of this EO. Our tests extended the insecticidal efficacy of this EO on other insect species, which are highly important in agricultural and public health settings. Additionally, the EO from *L. philippiana* has been previously assessed for its insecticidal efficacy against key stored product beetles *S. oryzae* (L.), *S. zeamais*, and *S. granarius* [13]. *L. philippiana* EO, mainly composed by methyl eugenol (61.38%) and safrole (14.76%), was tested on adults of *Sitophilus* spp., showing that the highest contact toxicity was achieved on *S. oryzae* at 4.0% (*v*/*w*). The same EO concentration also achieved a >80% antifeedant effect. The exposure to the EO led to a marked reduction in F_1_ emergence, which was at a maximum of 60% for *S. granarius* and *S. oryzae*, and 36% for *S. zeamais*. *Sitophilus* spp. have been found highly sensible to the fumigant toxicity and repellent effect of the above-mentioned EO. Similarly, the EO from *D. winteri* was successfully tested for its efficacy against stored product pests [39,40] as well as on the aphid *A. pisum* in deterrent bioassays [15]. Our work adds knowledge to the pool of information on the biological efficacy of this EO. Of note, we provided new information on the promising insecticidal efficacy of the EO from *A. boronioides,* which has not yet been studied for insecticidal activity.

As shown by our tests, the tested EOs showed promising insecticidal efficacy but, in many cases, it was not possible to identify the most effective one. Despite different chemical compositions, it could be hypothesized that very complex mixtures of several dozens of substances may be detrimental to their individual biological efficacies, most likely due to possible antagonistic relationships between the contained substances [34,35]. This phenomenon can, thus, result in the suppression of better biological efficacy of the major compounds. Although this hypothesis will have to be clarified in the tested EOs, this claim is supported by the work of other authors. *δ*-Cadinene can be mentioned as an example: this compound showed a major level of 45% in the tested EO from *P. uviferum*. As found by Govindarajan et al. [41], its level in the *Kadsura heteroclita* (Roxb.) EO was 18.3%, together with other major chemical components such as calarene (14.8%) and *δ*-4-carene (12.5%). This EO was tested for insecticidal activity on *Anopheles stephensi* Liston, *Aedes aegypti* (L.), and *C. quinquefasciatus* larvae, with LC_50_ values ranging from 103 to 122 µg mL^−1^. However, for the isolated substances *δ*-cadinene, calarene, and *δ*-4-carene, a higher efficacy was determined on *A. stephensi* (LC_50_ = 8, 12, and 16 µg mL^−1^, respectively), *A. aegypti* (LC_50_ = 9, 13, and 18 µg mL^−1^), and *C. quinquefasciatus* (LC_50_ = 10, 14, and 19 µg mL^−1^).

Despite the very promising insecticidal effects found for the tested EOs, we are very aware that further studies will be required to explore the effect of these EOs on nontarget organisms [42] to estimate the environmental impact of the areal application of botanical insecticides based on these EOs. Similarly, it will be necessary to study the impact of lethal and sublethal doses or concentrations on target species, considering that, as we know, even such applications can result in a significant reduction in subsequent population densities of target organisms; in practice, this can be utilized particularly to reduce the population density of flies and mosquitoes [43,44]. Additionally, possible ways of increasing the efficacy using nanoemulsions or encapsulation will be the subject of further studies [45].

## 4. Materials and Methods

### 4.1. Plant Collection

Plant materials used for isolation of the four EOs were collected in different areas of the Aysén region, Patagonia, Chile. Samples of *L. philippiana*, *P. uviferum,* and *D. winteri* were collected in December 2019, March 2019, and January 2019, respectively, in the locality of Valle Mirta (property Walwalun, La Junta, Aisén Region, Chile) at about 220 m a.s.l. (−43.88483222302501, −72.30618713473653). *A. boronioides* samples were collected in March 2019 in Puerto Ibañez along the carretera austral (Aisén Region, Chile) (−43.88483222302501,−72.30618713473653). Plants were identified by one of us (Daniela Santibañez Nieto) and deposited at the Herbarium of University of Antioquia. Selected parts of the plants used for EOs extraction were as follows: fresh green leaves, flowers, and resinous stems for *A. boronioides*; dry wood shavings for *P. uviferum*; dry leaves and flowering tops for *D. winteri;* fresh leaves and flowering tops for *L. philippiana*. Of note, the Patagonian plants subjected to hydrodistillation are quite rare and difficult to collect; due to the wet climate of the area, in situ drying is often difficult. For this reason, whenever possible, we processed the material as fresh and when not as dry material.

### 4.2. Isolation of Essential Oils

The EOs extraction from the selected plant materials were obtained through steam distillation. In detail, a 200 L alembic was filled with 50 L of water, and the plant material was placed over a metal mesh. When the alambique marked 60 °C, cold water was added to cool down the serpentine and allow the hydrolate to come out. For *L. philippiana*, 50 kg of leaves and stems were distilled to obtain 89 mL of EO, while, for *D. winteri*, 50 kg of leaves and stems were distilled to obtain 20 mL of EO. For *A. boronioides,* 100 kg of buds and some flowers were distilled to obtain 46 mL of EO, while for *P. uviferum,* 60 kg of wood shavings were distilled for the achievement of 45 mL of EO. Table 5 reports the yields (*v*/*w*) obtained for the four EOs calculated on a dry basis.

### 4.3. Essential Oils Chemical Characterization

The chemical characterization of the four EOs was performed using an Agilent 6890 N gas chromatograph equipped with a single-quadrupole 5973 N mass spectrometer and an auto-sampler 7863 (Agilent, Wilmington, DE, USA). The separation of EO components was achieved using an HP-5 MS capillary column (30 m, 0.25 mm i.d., 0.1 μm film thickness; 5% phenylmethylpolysiloxane), supplied by Agilent (Folsom, CA, USA). The analytical conditions and chromatogram analysis were the same as reported by Benelli et al. [45].

### 4.4. Insecticidal Activity

#### 4.4.1. Insecticidal Activity against Houseflies

Adults of *M. domestica* (males and females, 3–5 days old, from established laboratory colonies, >20 generations) were selected for the experiments. Contact toxicity of the four Patagonian EOs was evaluated through their topical application on the pronotum of *M. domestica* males and females. The four Patagonian EOs were prepared in acetone (Sigma-Aldrich, Schnelldorf, Germany) to obtain a concentration series (corresponding to the applied doses for females of 30, 50, 70, 100, and 150 µg adult^−1^ and for males of 10, 30, 50, 80, and 110 µg adult^−1^). Subsequently, 1 µL of EO was applied on each CO_2_-anesthetized fly through a micro-electric applicator. Acetone alone was used as the negative control. Then, the flies were moved to rearing containers sized 15 × 12 × 8 cm with a perforated lid (at 25 ± 1 °C, 70 ± 3% R.H., and 16:8 h (L:D)), containing their usual food. The experiment was replicated 4 times in total (20 insects per replication). Mortality was assessed 24 h after treatment. Insects failing to respond were considered dead.

#### 4.4.2. Insecticidal Activity against Moths

Larvae of *S. littoralis* (3rd instar, mean larval weight 12 ± 3 mg, from established laboratory colonies, >20 generations) were selected for the experiments. Contact toxicity of the four Patagonian EOs was evaluated through topical application on the dorsum of *S. littoralis* larvae. The EOs were prepared in acetone (Sigma-Aldrich, Germany) to obtain a concentration series (1 µL was applied using a micro-electric applicator to the dorsum, corresponding to the applied doses of 20, 40, 70, 90, and 110 µg larva^−1^). Acetone was used the negative control. Then, the larvae were moved to rearing containers sized 15 × 12 × 8 cm with a perforated lid (at 25 ± 1 °C, 70 ± 3% R.H., and 16:8 h (L:D)) and containing their usual food. The experiment was replicated 4 times in total (20 insects per replication). Mortality was assessed 24 h after treatment. Insects failing to respond were considered dead.

#### 4.4.3. Insecticidal Activity against Mosquitoes

*C. quinquefasciatus* larvae (3^rd^ instar) were exposed to four Patagonian EOs diluted in dimethyl sulfoxide (DMSO) relying on the WHO protocol [46] with minor changes by Pavela and Sedlak [47]; the tested concentrations were 20, 30, 50, 80, and 100 µL mL^−1^. Distilled water with the same amount of DMSO as that used for dissolving the EOs was the negative control. The experiments were carried out at 25 ± 1 °C, 70 ± 3% R.H., and 16:8 h (L:D). The experiments were replicated 4 times in total (25 insects per replication). Mortality was assessed 24 h after treatment. Insects failing to respond were considered dead.

### 4.5. Data Analysis

To calculate the EO lethal doses/concentrations on each target insect, we used a minimal series of at least 5 different doses/concentrations, which resulted in mortality rates in the range of 10–90%. Mortality was corrected using Abbott’s formula [48], and the lethal concentration values (LC_50_ and LC_90_) and associated 95% confidence limits for each treatment were estimated using probit analysis [49].

## 5. Conclusions

This study supports evidence of the insecticidal potential of EO obtained from ciprés *(P. uviferum)*, tepa *(L. philippiana)*, and canelo *(D. winteri)*, and demonstrates for the first time the insecticidal efficacy of the EO from paramela (*A. boronioides)*. The four Patagonian EOs are active against *M. domestica*, *S. littoralis,* and *C. quinquefasciatus* with promising LC_50,(90)_. However, it is not possible to establish which are the main responsible compounds for the biological activity, as the chemical composition is varied, and the different components could act synergistically or antagonistically. Notably, this study shows that these Patagonian plants can represent a local source of potential insecticidal products for the control of insects of medical and agricultural importance. Further studies are needed to explore the effects of these EOs on other target species, with special reference to the invasive moth pest *Spodoptera frugiperda* (J. E. Smith) [50], to determine their single components’ contribution to the insecticidal activity, and to evaluate their possible synergistic or antagonistic effect. Semi-field and field evaluation of the insecticidal activity of the most active botanical products will also be conducted.

## Figures and Tables

**Table 1:** Chemical composition of the essential oils from *Pilgerodendron uviferum* (ciprés), *Laureliopsis philippiana* (tepa), *Drimys winteri* (canelo), and *Adesmia boronioides* (paramela).

No	Component ^a^	RI ^b^	RI Lit. ^c^	Essential Oil				ID ^e^
				Ciprés % ^d^	Tepa %	Canelo %	Paramela%	
1	2-heptanone	893	889		Tr ^f^			RI, MS
2	2-heptanol	902	894		Tr			RI, MS
3	isobutyl isobutyrate	912	908		Tr			RI, MS
4	α-thujene	921	924		0.1 ± 0.0	0.1 ± 0.0	0.1 ± 0.0	RI, MS
5	α-pinene	926	932	Tr	1.0 ± 0.2	18.8 ± 2.8	7.0 ± 1.3	Std, RI, MS
6	ethyl tiglate	934	929				Tr	RI, MS
7	Camphene	939	946		Tr	0.5 ± 0.2	Tr	Std, RI, MS
8	thuja-2,4(10)-diene	945	953				0.5 ± 0.1	RI, MS
9	Benzaldehyde	955	952				Tr	Std, RI, MS
10	Sabinene	966	969		0.9 ± 0.2	1.8 ± 0.4	0.1 ± 0.0	Std, RI, MS
11	β-pinene	968	974		1.3 ± 0.3	21.5 ± 3.5	1.2 ± 0.3	Std, RI, MS
12	3-*p*-menthene	977	984				Tr	RI, MS
13	3-octanone	986	979			Tr		RI, MS
14	Myrcene	989	988		0.4 ± 0.2	0.9 ± 0.2		Std, RI, MS
15	2-pentyl-furan	990	990				0.1 ± 0.0	RI, MS
16	3-octanol	997	988			0.1 ± 0.0		RI, MS
17	α-phellandrene	1003	1002		3.0 ± 0.6	0.4 ± 0.1	0.2 ± 0.0	Std, RI, MS
18	δ-3-carene	1008	1008		0.1 ± 0.0	0.1 ± 0.0		Std, RI, MS
19	α-terpinene	1014	1014		0.1 ± 0.0	0.4 ± 0.1	0.2 ± 0.1	Std, RI, MS
20	*p*-cymene	1022	1020		1.1 ± 0.2	0.1 ± 0.0	1.5 ± 0.3	Std, RI, MS
21	limonene	1025	1024		0.8 ± 0.2	2.6 ± 0.5	2.1 ± 0.4	Std, RI, MS
22	1,8-cineole	1027	1026		8.5 ± 1.1	0.5 ± 0.1	0.1 ± 0.0	Std, RI, MS
23	(*Z*)-β-ocimene	1037	1032		0.1 ± 0.0	0.6 ± 0.2	Tr	Std, RI, MS
24	benzene acetaldehyde	1043	1036				Tr	RI, MS
25	(*E*)-β-ocimene	1047	1044		0.2 ± 0.0	Tr		Std, RI, MS
26	γ-terpinene	1055	1054		0.1 ± 0.0	0.7 ± 0.2	0.4 ± 0.1	Std, RI, MS
27	acetophenone	1065	1059				Tr	RI, MS
28	*cis*-linalool oxide	1071	1067		Tr			RI, MS
29	terpinolene	1085	1086		0.3 ± 0.1	0.3 ± 0.0	0.1 ± 0.0	Std, RI, MS
30	*p*-cymenene	1086	1089				0.1 ± 0.0	RI, MS
31	*trans*-linalool oxide	1087	1084		Tr			RI, MS
32	6-camphenone	1092	1095				Tr	RI, MS
33	2-nonanone	1094	1087		Tr			RI, MS
34	linalool	1100	1095		27.9 ± 3.1	2.8 ± 0.4	0.3 ± 0.1	Std, RI, MS
35	ethyl heptanoate	1101	1097				Tr	RI, MS
36	2-methyl butyl-2-methyl butyrate	1106	1100		Tr			RI, MS
37	1,3,8-*p*-menthatriene	1109	1108				Tr	RI, MS
38	*trans*-thujone	1113	1112				Tr	RI, MS
39	3-methyl-3-butenyl 3-methyl butanoate	1115	1112		Tr			RI, MS
40	α-campholenal	1122	1122				0.7 ± 0.2	RI, MS
41	*allo*-ocimene	1129	1128			Tr		RI, MS
42	*trans*-pinocarveol	1133	1135				0.2 ± 0.0	Std, RI, MS
43	camphor	1138	1141			0.1 ± 0.0		Std, RI, MS
44	*trans*-verbenol	1141	1140				0.1 ± 0.0	RI, MS
45	1,4-dimethyl-4-acetyl-1-cyclohexene	1147	1152				Tr	RI, MS
46	isobutyl hexanoate	1154	1149		Tr			RI, MS
47	*trans*-pinocamphone	1155	1158				Tr	RI, MS
48	pinocarvone	1157	1160				Tr	RI, MS
49	borneol	1161	1165			Tr		Std, RI, MS
50	δ-terpineol	1164	1162		Tr			RI, MS
51	*cis*-pinocamphone	1169	1172				Tr	RI, MS
52	ethyl benzoate	1170	1169				Tr	RI, MS
53	terpinen-4-ol	1172	1174		0.2 ± 0.0	0.6 ± 0.2	1.0 ± 0.2	Std, RI, MS
54	*p*-cymen-8-ol	1183	1179		Tr		0.1 ± 0.0	RI, MS
55	α-terpineol	1187	1186		2.1 ± 0.4	0.4 ± 0.1	0.3 ± 0.1	Std, RI, MS
56	myrtenal	1190	1195				0.2 ± 0.0	Std, RI, MS
57	methyl chavicol	1196	1195		Tr			RI, MS
58	verbenone	1204	1204				0.1 ± 0.0	RI, MS
59	*trans*-carveol	1218	1215				0.1 ± 0.0	RI, MS
60	carvone	1240	1239				0.1 ± 0.0	Std, RI, MS
61	bornyl acetate	1281	1287			Tr		Std, RI, MS
62	safrole	1284	1285		43.1 ± 3.9		Tr	RI, MS
63	theaspirane	1290	1298				Tr	RI, MS
64	indane derivative	1336					0.2 ± 0.0	MS
65	α-cubebene	1343	1345	Tr				RI, MS
66	eugenol	1355	1356		1.1	Tr		Std, RI, MS
67	α-copaene	1367	1374	6.1 ± 0.9			Tr	RI, MS
68	β-bourbonene	1376	1387		Tr			RI, MS
69	β-elemene	1385	1389	Tr		1.2 ± 0.3		Std, RI, MS
70	α-gurjunene	1400	1409			Tr		RI, MS
71	methyl eugenol	1406	1403		6.9 ± 1.1	Tr		RI, MS
72	(*E*)-caryophyllene	1409	1417	4.9 ± 0.8	0.2 ± 0.0	1.4 ± 0.3		Std, RI, MS
73	4,8-α-*epoxy*-caryophyllane	1412	1415	Tr				RI, MS
74	α-guaiene	1431	1437	0.2 ± 0.0				RI, MS
75	6,9-guaiadiene	1436	1442				4.3 ± 0.6	RI, MS
76	aromadendrene	1440	1439				0.7 ± 0.2	RI, MS
77	α-humulene	1443	1452	3.9 ± 0.7	Tr	0.2 ± 0.0		Std, RI, MS
78	*allo*-aromadendrene	1450	1458	Tr				RI, MS
79	(*E*)-β-farnesene	1452	1454			0.1 ± 0.0		Std, RI, MS
80	sesquisabinene	1456	1457			Tr		RI, MS
81	*trans*-cadina-1(6),4-diene	1466	1475	8.3 ± 1.2				RI, MS
82	γ-muurolene	1469	1478	0.8 ± 0.2				RI, MS
83	germacrene D	1471	1484		0.1 ± 0.0	Tr		RI, MS
84	β-selinene	1475	1489			0.1 ± 0.0	0.3 ± 0.1	RI, MS
85	β-dihydroagarofuran	1487	1496				1.8 ± 0.4	RI, MS
86	bicyclogermacrene	1487	1500		0.1 ± 0.0	2.6 ± 0.4		RI, MS
87	epizonarene	1491	1501	0.6 ± 0.2				RI, MS
88	α-muurolene	1493	1500	2.9 ± 0.6				RI, MS
89	esquel-6-en-9-one	1494					30.7 ± 3.1	MS
90	epishyobunone	1502	1498			0.2 ± 0.0		RI, MS
91	γ-cadinene	1505	1513	0.1 ± 0.0				RI, MS
92	*trans*-calamenene	1519	1521	3.4 ± 0.6				RI, MS
93	δ-cadinene	1521	1522	44.9 ± 2.9		0.1 ± 0.0		RI, MS
94	*trans*-cadina-1,4-diene	1525	1533	1.6 ± 0.3				RI, MS
95	γ-dehydro-*ar*-himachalene	1533	1530	0.6 ± 0.2				RI, MS
96	α-agarofuran	1533	1540				0.6 ± 0.2	RI, MS
97	α-calacorene	1535	1542	2.4 ± 0.2				RI, MS
98	furopelargone A	1536	1538				1.1 ± 0.3	RI, MS
99	hedycaryol	1543	1546	0.3 ± 0.1		18.2 ± 2.9		RI, MS
100	β-calacorene	1555	1564	0.1 ± 0.0				RI, MS
101	cryolan-8-ol	1558	1573	Tr				RI, MS
102	(*E*)-nerolidol	1562	1561			0.1 ± 0.0		Std, RI, MS
103	spathulenol	1567	1577		Tr	0.1 ± 0.0		RI, MS
104	caryophyllene oxide	1571	1583			Tr	0.7 ± 0.2	Std, RI, MS
105	gleenol	1578	1586	1.5 ± 0.2				RI, MS
106	*allo*-hedycariol	1579	1580			0.2 ± 0.0		RI, MS
107	furopelargone B	1583	1588				7.0 ± 1.1	RI, MS
108	esquel-7-en-9-one	1589					10.2 ± 1.4	MS
109	humulol	1591	1609	Tr				RI, MS
110	5-*epi*-7-*epi*-α-eudesmol	1596	1607			0.1 ± 0.0	0.4 ± 0.1	RI, MS
111	α-corocalene	1615	1622	0.2 ± 0.0				RI, MS
112	1-*epi*-cubenol	1620	1627	7.3 ± 0.9				RI, MS
113	10-*epi*-γ-eudesmol	1608	1622	0.4 ± 0.1		0.5 ± 0.1	2.2 ± 0.4	RI, MS
114	eremoligenol	1619	1629			0.6 ± 0.2		RI, MS
115	γ-eudesmol	1622	1630			6.6 ± 1.1		RI, MS
116	hinesol	1629	1640			0.3 ± 0.0		RI, MS
117	cubenol	1634	1645	5.4 ± 1.0				RI, MS
118	4-α-hydroxy-dihydro agarofuran	1634	1651				2.2 ± 0.5	RI, MS
119	α-muurolol	1639	1644	1.4 ± 0.3				RI, MS
120	β-eudesmol	1639	1649			5.2 ± 1.0		RI, MS
121	α-eudesmol	1643	1652	0.6 ± 0.2		6.3 ± 1.1		RI, MS
122	α-cadinol	1645	1652	0.3 ± 0.1				RI, MS
123	7-*epi*-α-eudesmol	1646	1662			0.2 ± 0.0		RI, MS
124	bulnesol	1656	1670			0.5 ± 0.1		RI, MS
125	cadalene	1665	1675	0.3 ± 0.1				RI, MS
126	kaurene	2039	2042			1.3 ± 0.3		RI, MS
127	*n*-heneicosane	2100	2100				0.1 ± 0.0	Std, RI, MS
128	*n*-tricosane	2300	2300				Tr	Std, RI, MS
	Total identified (%)			98.3	99.9	99.4	79.9	
	Grouped compounds (%)							
	Monoterpene hydrocarbons			Tr	18.0	48.7	13.6	
	Oxygenated monoterpenes				30.3	4.4	3.5	
	Sesquiterpene hydrocarbons			81.2	0.4	5.9	5.3	
	Oxygenated sesquiterpenes			17.2	Tr	39.0	57.2	
	Phenylpropanoids				51.1	Tr	Tr	
	Others				0.2	1.4	0.6	

^a^ The order of components is according to their elution from a HP-5MS column (30 m l. × 0.25 mm i.d., 0.1 mm f.t.). ^b^ Temperature-programmed linear retention index using a mixture of C7-C30 alkanes (Supelco, Bellefonte, CA). ^c^ Retention index value taken from Adams and/or NIST17 libraries. ^d^ Peak area percentage as the mean of three injections ± standard deviation. ^e^ Peak assignment method: Std, comparison of RT, RI, and MS with those of analytical standard (Sigma, Milan, Italy); RI, coherence of the experimentally determined RI with respect to those stored in ADAMS, NIST17, and FFNSC3 libraries; MS, mass fragmentation overlapping because of matching with ADAMS, WILEY275, FFNSC3, and NIST17 spectral libraries. ^f^ Traces, % <0.1.

**Table 2:** Insecticidal activity of the four essential oils from Patagonian plants against adults (females and males) of *Musca domestica; df = degrees of freedom, ns = not significant (p > 0.05)*.

Essential Oil	*M. domestica* Female						*M. domestica* Male					
	LC_50_ (µg adult^−1^)	C_I95_	LC_90_ (µg adult^−1^)	CI_95_	*χ^2^ (df = 3)*	*p*-Value	LC_50_ (µg adult^−1^)	CI_95_	LC_90_ (µg adult^−1^)	CI_95_	*χ^2^ (df = 3)*	*p*-Value
Canelo	76.7	60.1–96.5	296.5	259.7–312.5	1.766	0.622 ns	18.3	13.3–25.1	140.8	121.5–165.9	0.697	0.705 ns
Tepa	88.7	81.3–94.2	128.6	118.5–139.7	1.564	0.457 ns	24.6	18.5–29.7	119.3	89.4–139.7	1.096	0.777 ns
Paramela	65.2	51.7–78.1	195.1	156.5–220.1	2.583	0.273 ns	11.1	8.5–21.7	113.1	98.7–120.5	5.958	0.113 ns
Ciprés	68.6	58.2–75.8	183.7	152.5–211.1	1.782	0.257 ns	11.3	8.4–15.5	75.1	48.9–95.5	3.893	0.273 ns

**Table 3:** Insecticidal activity of the four essential oils from Patagonian plants against 3rd-instar larvae of *Spodoptera littoralis; df = degrees of freedom, ns = not significant (p > 0.05)*.

Essential Oil	LC_50_ (µg larva^−1^)	CI_95_	LC_90_ (µg larva^−1^)	CI_95_	*χ^2^ (df = 3)*	*p*-Value
Canelo	39.7	28.5–51.7	110.1	82.5–128.7	0.505	0.917 ns
Tepa	35.2	29.1–40.6	72.3	59.5–93.2	0.569	0.966 ns
Paramela	66.7	55.1–77.2	124.5	104.6–142.8	2.676	0.444 ns
Ciprés	33.8	26.5–41.7	106.3	89.7–127.6	1.861	0.761 ns

**Table 4:** Insecticidal activity of the four essential oils from Patagonian plants against 3rd-instar larvae of *Culex quinquefasciatus;* *df = degrees of freedom, ns = not significant (p > 0.05)*.

Essential Oil	LC_50_ (µL L^−1^)	CI_95_	LC_90_ (µL L^−1^)	CI_95_	*χ^2^*	*p*-Level	*df*
Canelo	48.6	33.5–62.8	111.2	98.5–126.9	3.129	0.536 ns	4
Tepa	52.2	39.8–61.1	81.5	71.8–92.7	1.452	0.325 ns	4
Paramela	77.3	72.5–82.1	110.6	101.5–124.3	1.762	0.623 ns	3
Ciprés	261.7	232.8–287.6	685.1	601.2–723.5	5.497	0.241 ns	4

**Table 5:** Yields of essential oils (mL/100 g) extracted from the four Patagonian plant species.

Plant Species	*L. philippiana*	*D. winteri*	*A. boronioides*	*P. uviferum*
Yield	0.06	0.04	0.04	0.07

## Data Availability

The data presented in this study are available on request from the corresponding authors.

## References

[1] Molares S., Ladio A. (2012). Mapuche perceptions and conservation of Andean. Nothofagus forests and their medicinal plants: A case study from a rural community in Patagonia, Argentina. Biodivers. Conserv..

[2] Molares S., Ladio A. (2014). Medicinal plants in the cultural landscape of a Mapuche-Tehuelche community in arid Argentine Patagonia: An eco-sensorial approach. J. Ethnobiol. Ethnomed..

[3] Martínez Crovetto R. (1980). Apuntes sobre la vegetación de los alrededores del Lago Cholila. Publicación Técnica De La Fac. De Cienc. Agrar..

[4] Silva F., Ullrich T., Hartman P., Medina H., Moraga L., Saini G. (2004). Plantas Medicinales de la región de Aysen-Chile. B Lat. Caribe Pl..

[5] Montes M., Wilkomirsky T. (1987). Medicina Tradicional Chilena.

[6] González S.B., Houghton P.J., Hoult J.R.S. (2003). The activity against leukocyte eicosanoid generation of essential oil and polar fractions of *Adesmia boronioides* Hook. f. Phytother. Res..

[7] González S.B., Ladio A.H., Gastaldi B., Silva Sofrás F.M., Mazzoni A., Sánchez G., Martinez J.L., Muñoz-Acevedo A., Rai M. (2018). Paramela (*Adesmia boronioides* Hook.f.): From popular uses to commercialization. Ethnobotany.

[8] Mølgaard P., Holler J.G., Asar B., Liberna I., Rosenbæk L.B., Jebjerg C.P., Jørgensen Jeanette L., Guzman A., Adsersen A., Simonsen H.T. (2011). Antimicrobial evaluation of Huilliche plant medicine used to treat wounds. J. Ethnopharmacol..

[9] Rodríguez R., Matthei O., Quezada M. (1983). Flora Arbórea de Chile.

[10] Bittner M., Aguilera M.A., Hernández V., Arbert C., Becerra J., Casanueva M.E. (2009). Fungistatic activity of essential oils extracted from *Peumus boldus* Mol., *Laureliopsis philippiana* (Looser) Schodde and *Laurelia sempervirens* (Ruiz & Pav.) Tul. (Chilean Monimiaceae). Chil. J. Agric. Res..

[11] Toledo D., Mutis A., Hormazabal E., Palma R., Parada M., Scheuermann E., Quiroz A. (2014). Chemical composition and antibacterial activity of *Laureliopsis philippiana* (Looser) essential oil. BLACPMA.

[12] Herrera-Rodríguez C., Ramírez-Mendoza C., Becerra-Morales I., Silva-Aguayo G., Urbina-Parra A., Figueroa-Cares I., Martínez-Bolaños L., Rodríguez-Maciel J.C., Lagunes-Tejeda A., Pastene-Navarrete E. (2015). Bioactivity of *Peumus boldus Molina*, *Laurelia sempervirens* (Ruiz & Pav.) Tul. and *Laureliopsis philippiana* (Looser) Schodde (Monimiaceae) essential oils against *Sitophilus zeamais* Motschulsky). Chil. J. Agric. Res..

[13] Norambuena C., Silva G., Urbina A., Figueroa I., Rodríguez-Maciel J.C. (2016). Insecticidal activity of *Laureliopsis philippiana* (Looser) Schodde (Atherospermataceae) essential oil against *Sitophilus* spp. (Coleoptera Curculionidae). Chil. J. Agric. Res..

[14] Cordero S., Abello L., Galvez F. (2017). Plantas Silvestres Comestibles y Medicinales de Chile y Otras Partes del Mundo.

[15] Zapata N., Lognay G., Smagghe G. (2010). Bioactivity of essential oils from leaves and bark of *Laurelia sempervirens* and *Drimys winteri* against *Acyrthosiphon pisum*. Pest. Manag..

[16] Tampe J., Espinoza J., Chacón-Fuentes M., Quiroz A., Rubilar M. (2020). Evaluation of *Drimys winteri* (Canelo) Essential Oil as Insecticide against *Acanthoscelides obtectus* (Coleoptera: Bruchidae) and *Aegorhinus superciliosus* (Coleoptera: Curculionidae). Insects.

[17] Cruz Madariaga G., Lara Aguilar A. (1981). Tipificación, Cambio de Estructura y Normas de Manejo para Ciprés de las Guaytecas (Pilgerodendron uvifera (D. Don) Florin.).

[18] Espinoza J., Urzúa A., Tampe J., Parra L., Quiroz A. (2016). Repellent activity of the essential oil from the heartwood of *Pilgerodendron uviferum* (D. Don) Florin against *Aegorhinus superciliosus* (Coleoptera: Curculionidae). Molecules.

[19] Espinoza J., Urzúa A., Bardehle L., Quiroz A., Echeverría J., González-Teuber M. (2018). Antifeedant effects of essential oil, extracts, and isolated sesquiterpenes from *Pilgerodendron uviferum* (D. Don) florin heartwood on red clover borer *Hylastinus obscurus* (Coleoptera: Curculionidae). Molecules.

[20] Di Giovanni F., Wilke A.B.B., Beier J.C., Pombi M., Mendoza-Roldan J.A., Desneux N., Canale A., Lucchi A., Dantas-Torres F., Otranto D. (2021). Parasitic strategies of arthropods of medical and veterinary importance. Entomol. Gen..

[21] Bass C., Denholm I., Williamson M.S., Nauen R. (2015). The global status of insect resistance to neonicotinoid insecticides. Pestic Biochem. Phys..

[22] Benelli G., Wilke A.B., Bloomquist J.R., Desneux N., Beier J.C. (2021). Overexposing mosquitoes to insecticides under global warming: A public health concern?. Sci. Total Environ..

[23] Yang X., Wei X., Yang J., Du T., Yin C., Fu B., Huang M., Liang J., Gong P., Liu S. (2021). Epitranscriptomic regulation of insecticide resistance. Sci. Adv..

[24] Malizia R.A., Cardell D.A., Molli J.S., González S., Guerra P.E., Grau R.J. (2000). Volatile constituents of leaf oils from the Cupressacea family: Part II. *Austrocedrus chilensis*, *Fitzroya cupressoides* and *Pilgerodendron uviferum* species growing in Argentina. J. Essent..

[25] Oyarzún M.L., Garbarino J.A. (1988). Sesquiterpenoids from *Pilgerodendron uvífera*. Phytochemistry.

[26] Madrid A., Godoy P., González S., Zaror L., Moller A., Werner E., Cuellar M., Villena J., Montenegro I. (2015). Chemical characterization and anti-oomycete activity of *Laureliopsis philippianna* essential oils against *Saprolegnia parasitica* and *S. australis*. Molecules.

[27] Barrero A.F., Herrador M.M., Arteaga P., Lara A., Cortes M. (2000). Chemical composition of the essential oil from *Drimys winteri* Forst. wood. J. Essent..

[28] Monsalvez M., Zapata N., Vargas M., Berti M., Bittner M., Hernández V. (2010). Antifungal effects of n-hexane extract and essential oil of Drimys winteri bark against Take-All disease. Ind. Crop. Prod..

[29] Muñoz O., Christen P., Cretton S., Barrero A.F., Lara A., Herrador M.M. (2011). Comparison of the essential oils of leaves and stem bark from two different populations of *Drimys winteri* a Chilean herbal medicine. Nat. Prod. Commun..

[30] Verdeguer M., García-Rellán D., Boira H., Pérez E., Gandolfo S., Blázquez M.A. (2011). Herbicidal Activity of *Peumus boldus* and *Drimys winterii* Essential Oils from Chile. Molecules.

[31] González S.B., Bandoni A.L., van Baren C., Lira P.D.L., Cerda-García-Rojas C.M., Joseph-Nathan P. (2002). Structure, conformation and absolute configuration of novel bisnorsesquiterpenes from the *Adesmia boronioides* essential oil. Tetrahedron.

[32] González S.B., Bandoni A.L., Van Baren C., Di Leo Lira P., Cerda-García-Rojas C.M., Joseph-Nathan P. (2004). The Essential Oil of the Aerial Parts of *Adesmia boronioides* Hook. F. J. Essent. Oil Res..

[33] Pavela R., Benelli G. (2016). Essential oils as ecofriendly biopesticides? Challenges and constraints. Trends Plant. Sci..

[34] Pavela R. (2015). Acute toxicity and synergistic and antagonistic effects of the aromatic compounds of some essential oils against *Culex quinquefasciatus* Say larvae. Parasitol. Res..

[35] Pavela R. (2014). Acute, synergistic and antagonistic effects of some aromatic compounds on the *Spodoptera littoralis* Boisd. (Lep., Noctuidae) larvae. Ind. Crop. Prod..

[36] Stepanycheva E., Petrova M., Chermenskaya T., Pavela R. (2019). Fumigant effect of essential oils on mortality and fertility of thrips *Frankliniella occidentalis* Perg. Environ. Sci. Pollut. Res..

[37] Pavela R., Žabka M., Bednář J., Tříska J., Vrchotová N. (2016). New knowledge for yield, composition and insecticidal activity of essential oils obtained from the aerial parts or seeds of fennel (*Foeniculum vulgare* Mill.). Ind. Crop. Prod..

[38] Espinoza J., Medina C., Aniñir W., Escobar-Bahamondes P., Ungerfeld E., Urzúa A., Quiroz A. (2021). Insecticidal, Repellent and Antifeedant Activity of Essential Oils from *Blepharocalyx cruckshanksii* (Hook. & Arn.) Nied. Leaves and *Pilgerodendron uviferum* (D. Don) Florin Heartwood against Horn Flies, *Haematobia irritans* (Diptera: Muscidae). Molecules.

[39] Paz C., Burgos V., Iturra A., Rebolledo R., Ortiz L., Baggio R., Cespedes-acuña C.L. (2018). Industrial Crops & Products Assessment of insecticidal responses of extracts and compounds of *Drimys winteri, Lobelia tupa*, *Viola portalesia* and *Vestia foetida* against the granary weevil *Sitophilus granarius*. Ind. Crop. Prod..

[40] Zapata N., Smagghe G. (2010). Repellency and toxicity of essential oils from the leaves and bark of *Laurelia sempervirens* and *Drimys winteri* against *Tribolium castaneum*. Ind. Crop. Prod..

[41] Govindarajan M., Rajeswary M., Benelli G. (2016). δ-Cadinene, calarene and δ-4-carene from *Kadsura heteroclita* essential oil as novel larvicides against malaria, dengue and filariasis mosquitoes. Comb. Chem High. Throughput Screen.

[42] Pavela R., Morshedloo M.R., Mumivand H., Khorsand G.J., Karami A., Maggi F., Desneux N., Benelli G. (2020). Phenolic monoterpene-rich essential oils from Apiaceae and Lamiaceae species: Insecticidal activity and safety evaluation on non-target earthworms. Entomol. Gen..

[43] Sánchez-Gómez S., Pagán R., Pavela R., Mazzara E., Spinozzi E., Marinelli O., Zeppa L., Morshedloog M.R., Maggi F., Canale A. (2022). Lethal and sublethal effects of essential oil-loaded zein nanocapsules on a zoonotic disease vector mosquito, and their non-target impact. Ind. Crop. Prod..

[44] Benelli G., Pavela R., Giordani C., Casettari L., Curzi G., Cappellacci L., Petrelli R., Maggi F. (2018). Acute and sub-lethal toxicity of eight essential oils of commercial interest against the filariasis mosquito *Culex quinquefasciatus* and the housefly *Musca domestica*. Ind. Crop. Prod..

[45] Benelli G., Pavoni L., Zeni V., Ricciardi R., Cosci F., Cacopardo G., Gendusa S., Spinozzi E., Petrelli R., Cappellacci L. (2020). Developing a highly stable *Carlina acaulis* essential oil nanoemulsion for managing *Lobesia botrana*. Nanomaterials.

[46] WHO (1996). Report of the WHO Informal Consultation on the Evaluation and Testing of Insecticides.

[47] Pavela R., Sedlák P. (2018). Post-application temperature as a factor influencing the insecticidal activity of essential oil from *Thymus vulgaris*. Ind. Crop. Prod..

[48] Abbott W.S. (1925). A method of computing the effectiveness of an insecticide. J. Econ. Entomol..

[49] Finney D.J. (1971). Statistical logic in the monitoring of reactions to therapeutic drugs. Methods Inf. Med..

[50] Kenis M., Benelli G., Biondi A., Calatayud P.A., Day R., Desneux N., Harrison R.D., Kriticos D., Rwomushana I., van den Berg J. (2022). Invasiveness, biology, ecology, and management of the fall armyworm, *Spodoptera frugiperda*. Entomol. Gen..

